# The impact of residential greenness on psychological distress among Hurricane Katrina survivors

**DOI:** 10.1371/journal.pone.0285510

**Published:** 2023-05-11

**Authors:** Kate Burrows, Kelvin C. Fong, Sarah R. Lowe, Elizabeth Fussell, Michelle L. Bell

**Affiliations:** 1 Institute at Brown for Environment and Society, Brown University, Providence, RI, United States of America; 2 Department of Earth and Environmental Sciences, Dalhousie University, Halifax, NS, Canada; 3 Department of Social and Behavioral Sciences, School of Public Health, Yale University, New Haven, CT, United States of America; 4 School of the Environment, Yale University, New Haven, CT, United States of America; Drexel University, UNITED STATES

## Abstract

Residential greenness may support mental health among disaster-affected populations; however, changes in residential greenness may disrupt survivors’ sense of place. We obtained one pre- and three post-disaster psychological distress scores (Kessler [K]-6) from a cohort (n = 229) of low-income mothers who survived Hurricane Katrina in New Orleans, Louisiana, USA. Greenness was assessed using average growing season Normalized Difference Vegetation Index (NDVI) and Enhanced Vegetation Index (EVI) in the 300 m around participants’ homes at each time point. We used multivariable logistic regressions to evaluate two hypotheses: 1) that cross-sectional greenness (above vs. below median) was associated with reduced psychological distress (K6≥5); and 2) that changes in residential greenness were associated with adverse mental health. When using EVI, we found that a change in level of greenness (*i*.*e*., from high to low [high-low], or from low to high [low-high] greenness, comparing pre- and post-Katrina neighborhoods) was associated with increased odds of distress at the first post-storm survey, compared to moving between or staying within low greenness neighborhoods (low-high odds ratio [OR] = 3.48; 95% confidence interval [CI] = 1.40, 8.62 and high-low OR = 2.60; 95% CI: 1.05, 6.42). Results for NDVI were not statistically significant. More research is needed to characterize how residential greenness may impact the health of disaster survivors, and how these associations may change over time.

## 1. Introduction

Hurricane Katrina made landfall in New Orleans, Louisiana, United States, on August 29, 2005, and had catastrophic impacts, including more than 1,500 fatalities and $40–50 billion in monetary losses [1]. Such environmental disasters are potentially traumatic events with long-lasting health issues for survivors. A robust body of literature has shown that Hurricane Katrina was associated with adverse mental [2] and physical [3, 4] health outcomes along with extensive impacts on survivors’ economic wellbeing [5], employment [6], and residential mobility [7]. Many of these impacts varied across gender [8], race [9], and socioeconomic status [10]. Beyond individual-level disparities, additional research on disaster survivors has suggested that neighborhood-level social features, such as social and economic capital, are associated with lower risk of mortality and morbidity among survivors [11–14]. However, there is very limited research on the potential impact of neighborhood-level *environmental* features on the health outcomes of disaster survivors.

In this study, we focused on residential greenness, which refers to vegetation in the neighborhood or area surrounding an individual’s home. This may include wooded areas, parks, gardens, or agricultural areas, in addition to vegetated vacant lots. Research has found that residential greenness is associated with a range of health outcomes, including reductions in mortality, higher birth weight and reduced risk of low birth weight [15], and improved mental health [16, 17]. A number of causal pathways linking greenness and mental health have been suggested, with most falling into four categories: increased social interaction, increased physical activity, reduction of harms associated with air and noise pollution and heat [18], and biophilia, which refers to an intrinsic human desire to spend time in nature [19].

The mental health benefits of greenness for disaster survivors may therefore be similar to that of the general population, with greener post-disaster neighborhoods being associated with improved mental health [20]. However, critical to the experience of many disaster survivors is a *change* in residential greenness. After environmental disasters, an individual’s residential greenness may change because: (1) neighborhoods may change (e.g., hurricanes can alter vegetation coverage) [21] or (2) they may relocate to new neighborhoods with different greenspace patterns than the original neighborhood. For survivors, a change in residential greenness may cause disruptions to place attachment, which refers to the emotional connection between people and a particular place [22]. Place attachment has been cited as an influential factor in post-disaster mental health [23], recovery [24, 25], mobility decisions [26, 27], and religious experiences [28]. Theoretical understandings of place attachment include both physical and natural environments, but historically the focus has been placed on *social* constructions of place [22]. However, especially in the context of global climate change, scholars are increasingly emphasizing the role of the natural environment as a critical dimension of place attachment [29–31]. Therefore, while residential greenness may be beneficial to mental health, a *change* in neighborhood greenness may be associated with worsened mental health through disruptions to place attachment.

In this study, we focused on a unique cohort of lower-income parents who resided in the Greater New Orleans area and reported living in areas that were affected by Hurricane Katrina in 2005 [32]. We investigated the association between residential greenness and mental health using Normalized Difference Vegetation Index (NDVI) and Enhanced Vegetation Index (EVI) in the 300m surrounding respondents’ residences and compared changes in residential greenness across three different time periods after Katrina (2006–2018).

We tested two hypotheses: 1) residential greenness in post-disaster location is positively associated with mental health; and 2) changes in residential greenness are associated with adverse mental health in that they may pose disruptions to place attachment. By leveraging pre-disaster data, this is the first study to our knowledge to examine whether *changes* in residential greenness over time are associated with mental health outcomes among disaster survivors. Further, the use of a longitudinal cohort is a notable advancement in the broader study of greenness and mental health, which has largely relied on cross-sectional data [33]. By controlling for baseline mental health, this study design also attempted to address questions of self-selection, where individuals who have better mental health may be more likely to reside in greener neighborhoods [33, 34]. Following a cohort of hurricane survivors over nearly 15 years also helped to elucidate temporal patterns in the relationship between greenness and mental health that cannot be measured by cross-sectional designs, thus contributing to the emerging literature on the role of neighborhood environmental characteristics in supporting health after disasters.

## 2. Methods

### 2.1 Resilience in survivors of Katrina survey

We used data from the Resilience in Survivors of Katrina (RISK) Survey. This cohort was originally assembled in 2004–2005 as part of a longitudinal study to assess the effect of a community college educational intervention among low-income parents in New Orleans [35]. The original study subjects were parents, ages 19–34 years, who were enrolled or planning to enroll in a community college, had a household income under 200% of the federal poverty level, and had a high school diploma or equivalent [35]. After Hurricane Katrina, the project was redesigned to study the impact of the disaster on the long-term health, well-being, and socioeconomic status of participants. The participants of the RISK cohort comprise an important and high-risk group of disaster survivors. In addition to the baseline data collected before Hurricane Katrina (time 0 [T0] in 2004–2005), three post-hurricane follow-up surveys have been conducted: T1 in 2006–2007, T2 in 2009–2010, and T3 in 2016–2018. The original cohort consisted of 1,019 participants. A total of 711 participants completed T1 (69.8%), 752 participants completed T2 (73.8%), and 716 (70.2%) participants completed T3. Nearly all participants in the original sample identified as female [36].

The outcome of interest was post-disaster general psychological distress, as measured by the Kessler’s K6 scale, which ranges from 0–24 [37]. We selected K6 as it is a broad screening tool that captures a range of psychological disorders, rather than screening for a specific disorder (such as the Patient Health Questionnaire [PHQ] or the Post-Traumatic Stress Disorder Checklist-S [PCL-S]) [38]. Further, the K6 is widely used in disaster research [39]. In our primary analysis we categorized K6 based on whether scores fell above or below the cut-off for moderate, but still clinically significant, psychological distress (scores ≥5, hereafter the outcome is referred to simply as “psychological distress”) [40]. The longitudinal nature of our data allowed us to control for K6 over time, including pre-disaster (baseline) data.

We adjusted for possible confounders including age at baseline, race (Black, white, other), self-reported social support at the time of each survey (measured using an abbreviated form of the Social Provisions Scale [SPS] ranging from 1–4) [41] and whether a respondent’s household was receiving benefits (yes vs. no) at the time of each survey (measured based on whether a person reported their household receiving one or more of the following benefits: unemployment, disability [Social Security Disability Insurance and Supplemental Security Income], welfare [cash assistance or Temporary Assistance for Needy Families, or Supplemental Nutrition Assistance Program]). Receiving any of these benefits was used as a proxy for household socioeconomic status. For post-Katrina time periods (T1, T2, and T3), we included the level of housing damage associated with the storm (minor or no damage vs. major damage or destroyed) as a covariate in our models. We also adjusted for whether a person was living in New Orleans at the time of each survey (yes vs. no), which previous research has identified as associated with mental health. For example, Fussell and Lowe [42] found that among RISK participants, return to New Orleans was associated with lower K6 scores compared to those who had not returned. Last, we controlled for distance between residential locations at each the time of each survey to adjust for the role of residential mobility, which may be associated with mental health after environmental disasters [43]. To further account for mobility, we also conducted a sensitivity analysis in which we used a binary indicator for whether a person reported moving in lieu of distance, to help ensure that we were assessing the impact of changes in greenness on mental health, not simply the impact of relocation.

### 2.2 Residential greenness

To calculate residential greenness, we obtained remote sensing measurements from the Moderate Resolution Imaging Spectrometer (MODIS) aboard the Terra satellite [44]. For data retrieval, we used rgee, an R package that implements Google Earth Engine’s API [45]. We created a 300m buffer around residential addresses and computed the mean EVI and NDVI within each buffer across the growing season (June-August) in the year of their interview. We selected a 300m buffer size as it is commonly used in studies related to greenness and mental health [46], and because it reflects the World Health Organization guideline that all people should have a large public green space within 300 m from their home (approximately a 5-minute walk) [47]. While other data sources may provide higher spatial resolution (e.g., Landsat), studies show that different satellite sensors produce relatively similar estimates of NDVI and EVI [48, 49]. We chose to use a growing season average to adjust for seasonality and to capture the general greenness of the area.

Each participant was assigned NDVI and EVI values for residential greenness based on the GPS coordinates for their home address for each survey (T0, T1, T2, and T3). Respondents were categorized as living in areas with low (below median) or high (above median) residential greenness at each time point. In our analyses, we assessed both the association between cross-sectional greenness and mental health at each survey and the *change* in residential greenness across waves and mental health at each survey (*i*.*e*., whether a person resided in a low greenness area at the first wave, and then a high greenness area in the second [low-high], between low areas [low-low], between high areas [high-high], or from high to low areas [high-low]). We also conducted sensitivity analyses using 500m and 1000m buffers.

### 2.3 Neighborhood concentrated disadvantage

Residential greenness is often associated with other neighborhood-level characteristics, including level of income or racial and ethnic factors [50], which could potentially impact mental health after disasters. To help account for these other neighborhood-level characteristics, we estimated the census tract-level Neighborhood Concentrated Disadvantage (NCD). The NCD index was originally developed by Sampson, Raudenbush [51], and is calculated using tract level data on: 1) percent of individuals below the poverty line, 2) percent of individuals on public assistance, 3) percent female-headed households, 4) percent unemployed, and 5) percent less than age 18 years. After obtaining these values at the census tract level, we Z-score transformed each of the five indicators, and created a composite score for NCD by taking the average of the transformed indicators [52]. Higher values of NCD correspond to higher neighborhood disadvantage. We used data from the 2000 Decennial Census for T0 (pre-Katrina), and for post-Katrina waves, we used data from the American Community Survey (ACS) 5-year estimates (T1: 2005–2009; T2: 2009–2013; T3: 2015–2019). Comparing across non-overlapping 5-year estimates and decennial census data is considered acceptable according to Census Bureau guidelines [53].

As we did for residential greenness, we separately modeled the cross-sectional NCD as well as the neighborhood change in NCD between survey waves (e.g., moving from a low NCD area to a high NCD area [low-high], moving between two low NCD areas [low-low], moving between a high and low NCD area [high-low] or between two high NCD areas [high-high]) as a covariate at each time point.

### 2.4 Statistical analyses

We conducted a complete case analysis on the 229 women with complete data for all variables of interest at all time points. Nearly all RISK participants identified as women (92.4% of baseline sample), and those who identified as men were dropped in the third wave [36]. As a preliminary assessment of the dataset, we assessed whether K6 scores were significantly different at baseline among those included in this analysis (*i*.*e*., those with complete data, n = 229) compared to those who were excluded (n = 790).

To test our first hypothesis (that residential greenness in post-disaster location was positively associated with mental health), we applied separate generalized logistic regressions to assess whether K6 scores were significantly associated with neighborhood greenness at T0, T1, T2, and T3. To test our second hypothesis (that changes in residential greenness were negatively associated with mental health), we modeled whether change in neighborhood-level residential greenness (i.e., low-low, low-high, high-low, high-high) was associated with psychological distress at each time point (e.g., whether residing in a neighborhood with low residential greenness at T0 but a neighborhood with high residential greenness at T1 was associated with psychological distress at T1).

All models were adjusted for sociodemographic characteristics (age at baseline, race, perceived social support at the time of each survey, and whether a person received benefits at the time of each survey), Katrina exposure (Katrina-related housing damage as assessed at T1), neighborhood characteristics (NCD and whether a person lived in New Orleans or not at the time of each post-disaster survey), and the distance of residential relocation between each wave. We also adjusted for psychological distress at the previous survey. To account for potential clustering, we calculated robust standard errors using Huber-White estimates for all models. Statistical analyses were performed in RStudio (Version 1.4.1106). Statistical significance was assessed at the α = 0.05 level.

## 3. Results

### 3.1 Summary statistics

Baseline psychological distress was not significantly different (t-test, p = 0.261) between the 229 participants who were included in the study (mean K6 = 4.68 [SD = 3.69]) and the 790 participants who were excluded from the study based on missing data for at least one variable at one time point (mean K6 = 5.01 [SD = 4.26]). Full summary statistics for included participants are shown in Table 1. K6 scores show that psychological distress increased in the first years after Katrina (mean score of 6.10 [SD = 4.77] at T1). K6 scores were lower at T2 (mean 5.61 [SD = 4.70]) and T3 (mean 5.86 [SD = 4.76]) than they were in the first wave (T1), but remained higher than at baseline (mean 4.68 [SD = 3.68]).

**Table 1:** Summary statistics at each timepoint of data collection for participants with complete data (n = 229).

Category		Survey			
		Baseline (pre-hurricane, 2004–2005)	T1 (1-2y post hurricane)	T2 (4-5y post hurricane)	T3 (11-13y post hurricane)
Psychological distress	**K6 Score (0–24)** ^**i**^				
	Mean (SD)	4.68 (3.68)	6.10 (4.77)	5.61 (4.70)	5.86 (4.76)
	Median [Min, Max]	4.00 [0, 24.0]	5.00 [0, 24.0]	4.00 [0, 20.0]	5.00 [0, 22.0]
	**Psychological Distress (K6 ≥ 5)**				
	No distress	128 (55.9%)	102 (44.5%)	119 (52.0%)	107 (46.7%)
	Psychological distress	101 (44.1%)	127 (55.5%)	110 (48.0%)	122 (53.3%)
Socio-demographics	**Baseline Age (years)**				
	Mean (SD)	25.20 (4.46)	-	-	-
	Median [Min, Max]	25.0 [18.0, 35.0]	-	-	-
	**Race**				
	White	22.0 (9.6%)	-	-	-
	Black	196 (85.6%)	-	-	-
	Other	11.0 (4.8%)	-	-	-
	**Generalized Support (1–4)** ^**ii**^				
	Mean (SD)	3.20 (0.453)	3.20 (0.483)	3.19 (0.424)	3.22 (0.523)
	Median [Min, Max]	3.13 [1.38, 4.00]	3.13 [1.25, 4.00]	3.13 [1.75, 4.00]	3.25 [1.75, 4.00]
	**Received benefits**				
	No	56.0 (24.5%)	184 (80.3%)	93.0 (40.6%)	127 (55.5%)
	Yes	173 (75.5%)	45.0 (19.7%)	136 (59.4%)	102 (44.5%)
Damage Associated with Katrina	**Level of Damage to Housing**				
	Minor or no damage	-	137 (59.8%)	-	-
	Major damage or Destroyed	-	92.0 (40.2%)	-	-
Neighborhood Features	**Distance of Relocation (km) Between Surveys**				
	Mean (SD)	-	269 (408)	189 (425)	138 (344)
	Median [Min, Max]	-	11.4 [0, 3030]	6.29 [0, 3030]	6.29 [0, 2430]
	**Relocated At Least Once Between Surveys**				
	No	-	2 (0.87%)	5 (2.22%)	38 (16.6%)
	Yes	-	227 (99.1%)	224 (97.8%)	191 (83.4%)
	**Currently living in New Orleans**				
	No	-	112 (48.9%)	77 (33.6%)	87.0 (38.0%)
	Yes	-	117 (51.1%)	152 (66.4%)	142 (62.0%)
	**Neighborhood Concentrated Disadvantage (NCD)** ^**iii**^				
	Mean (SD)	0.570 (0.719)	0.450 (0.664)	0.438 (0.707)	0.406 (0.645)
	Median [Min, Max]	0.507 [-1.10, 3.26]	0.410 [-0.953, 2.39]	0.307 [-1.10, 4.60]	0.317 [-0.824, 3.57]
	**Change in NCD Between Surveys** ^**iv**^				
	Low-low	-	74.0 (32.3%)	81.0 (35.4%)	62.0 (27.1%)
	High-low	-	41.0 (17.9%)	34.0 (14.8%)	53.0 (23.1%)
	Low-high	-	43.0 (18.8%)	34.0 (14.8%)	53.0 (23.1%)
	High-high	-	71.0 (31.0%)	80.0 (34.9%)	61.0 (26.6%)
	**Greenness (NDVI)** ^**v**^				
	Mean (SD)	0.475 (0.123)	0.453 (0.122)	0.455 (0.123)	0.477 (0.127)
	Median [Min, Max]	0.473 [0.143, 0.785]	0.463 [0.120, 0.753]	0.446 [0.124, 0.792]	0.465 [0.105, 0.813]
	**Change in Greenness Between Surveys (NDVI)** ^**vi**^				
	High-high	-	59.0 (25.8%)	56.0 (24.5%)	60.0 (26.2%)
	Low-high	-	55.0 (24.0%)	58.0 (25.3%)	54.0 (23.6%)
	High-low	-	55.0 (24.0%)	58.0 (25.3%)	54.0 (23.6%)
	Low-low	-	60.0 (26.2%)	57.0 (24.9%)	61.0 (26.6%)
	**Greenness (EVI)** ^**v**^				
	Mean (SD)	0.306 (0.083)	0.316 (0.081)	0.318 (0.086)	0.323 (0.085)
	Median [Min, Max]	0.302 [0.091, 0.52]	0.325 [0.081, 0.484]	0.319 [0.085, 0.552]	0.329 [0.079, 0.552]
	**Change in Greenness Between Surveys (EVI)** ^**vi**^				
	High-high	-	58 (25.3%)	61 (26.6%)	61 (26.6%)
	Low-high	-	56 (24.5%)	51 (22.3%)	53 (23.1%)
	High-low	-	56 (24.5%)	53 (23.1%)	51 (22.3%)
	Low-low	-	59 (25.8%)	64 (27.9%)	64 (27.9%)

Notes

i. Higher K6 scores (0–24) indicate higher levels of psychological distress.

ii. Higher values (1–4) indicate higher levels of perceived social support.

iii. Higher NCD scores indicate areas with more neighborhood-level disadvantage.

iv. Indicates whether a person moved between two low NCD areas (low-low), from a low NCD area to a high NCD area (low-high), between two high NCD areas (high-high), or between a high and low NCD area (high-low) between the current wave and the previous wave.

v. Higher NDVI and EVI values indicate areas with more residential greenness.

vi. Indicates whether a person moved between two low greenness areas (low-low), from a low greenness area to a high greenness area (low-high), between two high greenness areas (high-high), or between a high and low greenness area (high-low) between the current wave and the previous wave.

Residential greenness stayed relatively consistent across survey waves: at baseline (2004–2005), average EVI was 0.306 (SD = 0.083), and NDVI was 0.475 (SD = 0.123); at T1 (1–2 years post hurricane), EVI was 0.316 (SD = 0.081) and NDVI was 0.453 (SD = 0.122); at T2 (4–5 years post hurricane), EVI was 0.318 (SD = 0.086) and NDVI was 0.455 (SD = 0.123); and at T3 (11–13 years post hurricane), EVI was 0.323 (SD = 0.085) NDVI was 0.477 (SD = 0.127). However, NCD scores decreased across survey waves: the average NCD score was 0.570 (SD = 0.719) at baseline; 0.450 (0.664) at T1; 0.438 (0.707) at T2; and 0.406 (0.645) at T3, reflecting an overall improvement in participants’ neighborhood disadvantage level based on NCD. Most participants relocated between survey waves; between baseline and T1, 99.1% of participants relocated at least once. The percent of participants who relocated between survey waves decreased over time but remained high, with 97.8% of participants relocating between T1 and T2, and 83.4% relocating between T2 and T3.

We tested for correlations across predictors using Spearman’s rank correlations (to account for non-linear relationships) at each of the three post-Katrina time periods. Correlation was limited (all *r* < 0.40) indicating that it was appropriate to include all of the variables in the models.

### 3.2 Hypothesis 1: Association between greenness and psychological distress

**Fig 1:**
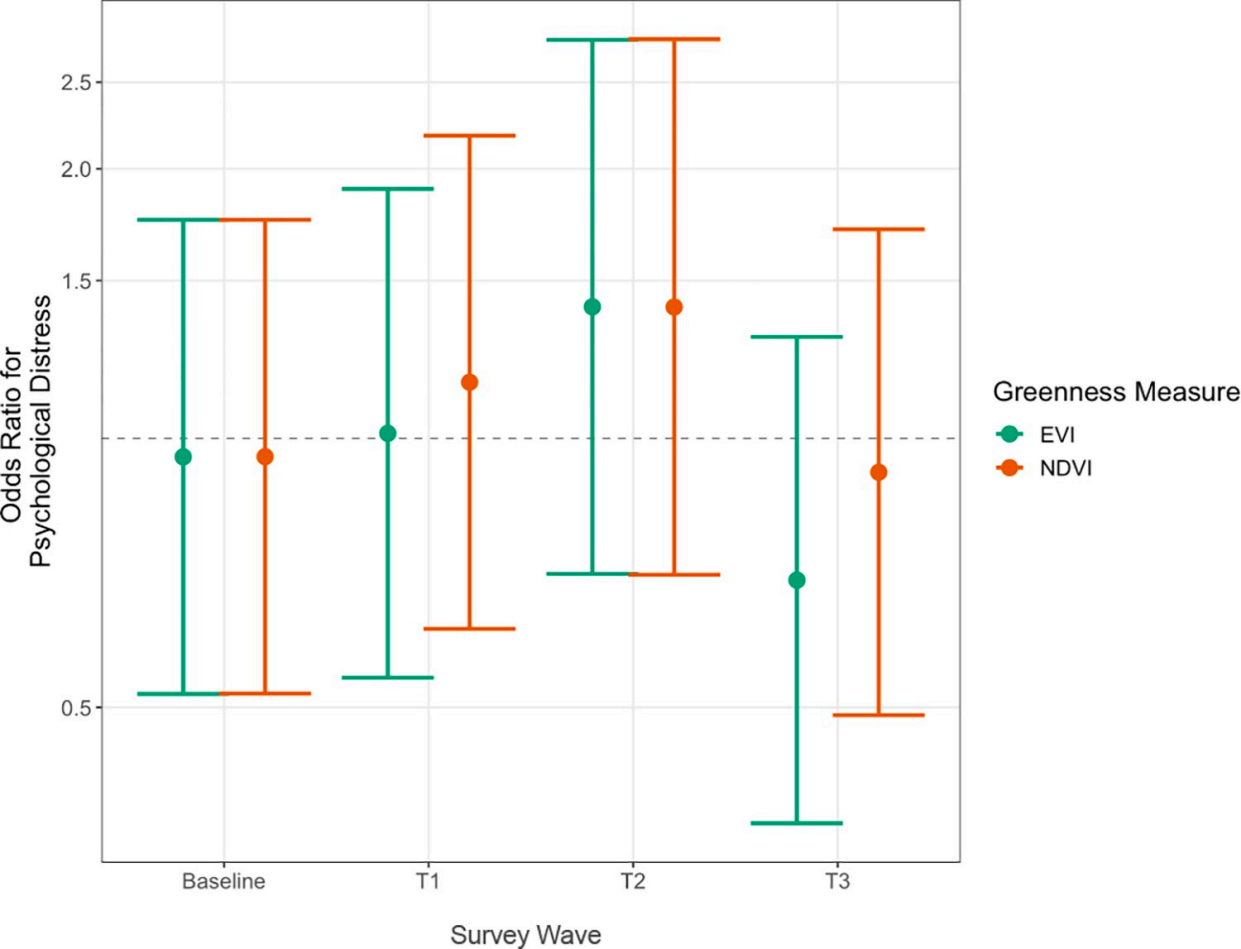
Fully adjusted cross-sectional associations between residential greenness (high vs. low greenness areas, measured by NDVI and EVI 300m buffers) and odds of psychological distress. Distress (K6 ≥ 5) was evaluated at each survey wave (n = 229). Results are from multivariable logistic regressions (β [95% CI]) with robust standard errors calculated using Huber-White estimates for all models.

We did not find evidence to support our first hypothesis: results from our analysis show that cross-sectional residential greenness (living in areas of high vs. low residential greenness as measured by NDVI and EVI) was not significantly associated with psychological distress in any of the surveys (results presented in Fig 1, full results shown in S1 [EVI] and S2 Tables [NDVI] in **S1 Appendix**). The odds of reporting psychological distress were not statistically different between participants living in high and low greenness neighborhoods at T0 (OR: 0.91 [95% CI: 0.50–1.63]), T1 (OR: 1.01 [95% CI: 0.54–1.90]), T2 (OR: 1.40 [95% CI: 0.71–2.79]), T3 (OR: 0.69 [95% CI: 0.37–1.3]) when using EVI. Results were consistent for NDVI: T0 (OR: 0.96 [95% CI: 0.54–1.72]), T1 (OR: 1.15 [95% CI: 0.61–2.18]), T2 (OR: 1.40 [95% CI: 0.70–2.79]), and T3 (OR: 0.92 [95% CI: 0.49–1.71]).

### 3.3 Hypothesis 2: Association between changes in greenness and psychological distress

**Fig 2:**
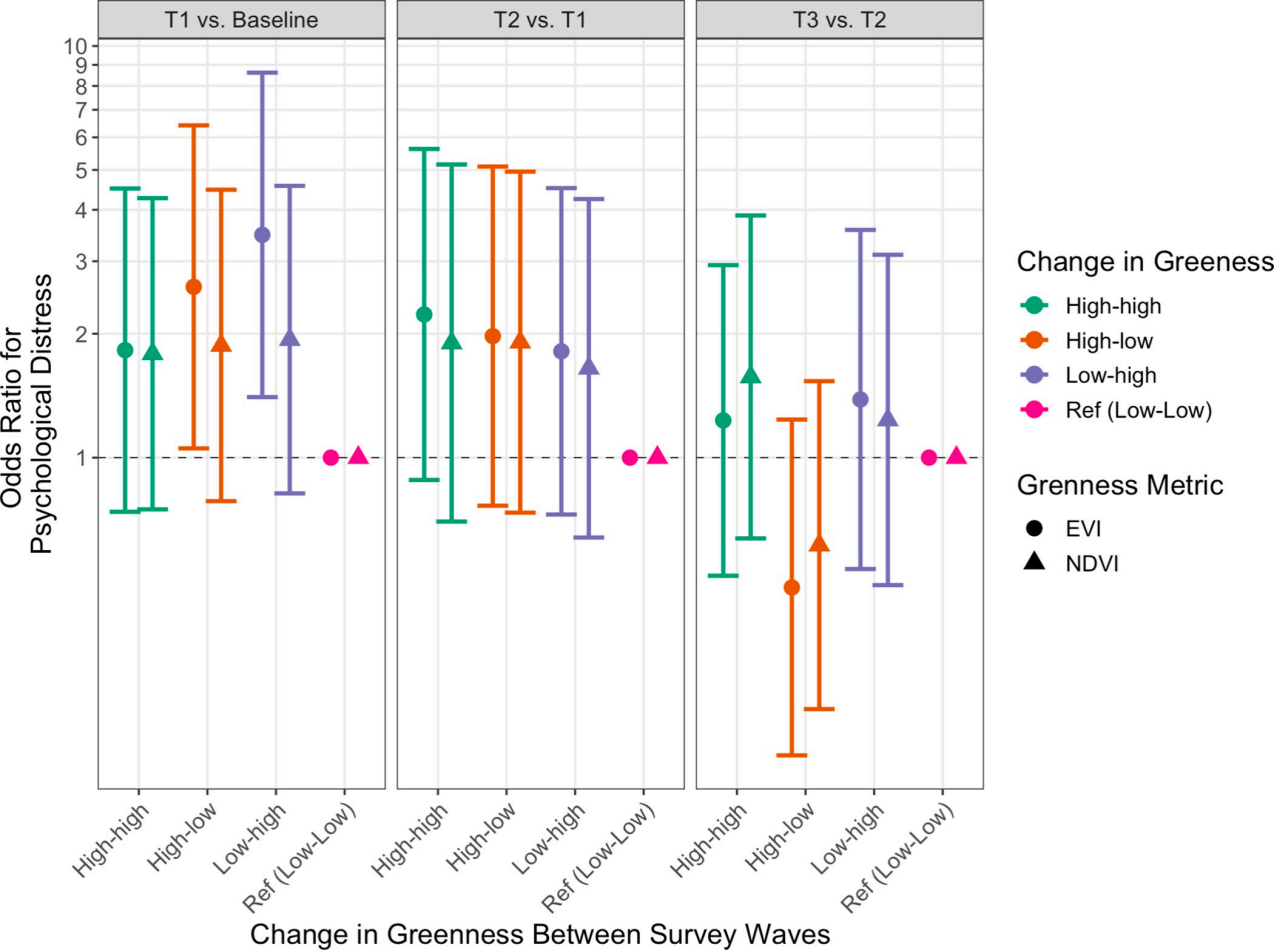
Fully adjusted association between change in greenness (NDVI and EVI) between survey waves and odds of psychological distress. Distress (K6 ≥ 5) was evaluated at each survey wave (n = 229). Results from logistic regressions (β [95% CI]) with robust standard errors calculated using Huber-White estimates for all models.

We next tested our second hypothesis by leveraging the longitudinal study design to assess whether *change* in residential greenness between survey waves (*i*.*e*., comparing T1 to T0 and T2 to T1, etc.) was associated with the odds of reporting psychological distress at the later wave (controlling for psychological distress at the previous wave, sociodemographic characteristics, Katrina exposure, and neighborhood characteristics). Results are presented in Fig 2 (full models shown in S3 [EVI] and S4 Tables [NDVI] in S1 Appendix).

Based on EVI, we found evidence to support our hypothesis that participants whose neighborhood greenness changed between baseline and T1 from low to high (OR: 3.48, 95% CI: 1.40–8.62) or from high to low (OR: 2.60, 95% CI: 1.05–6.42) had increased odds of psychological distress at T1 compared to participants whose greenness was low at baseline and remained low at T1. However, when comparing greenness between later waves (T2 vs. T1 and T3 vs. T2), we did not find that greenness significantly impacted the odds of distress. Change in residential greenness was not significantly associated with psychological distress at any of the three post-Katrina surveys (T1, T2, T3) when using NDVI.

As a sensitivity analysis we used different buffer sizes (500m and 1000m) around participants’ residential addressees (S1 Fig in **S1 Appendix**). The results for NDVI were consistent across buffers–change in residential greenness was not significantly associated with psychological distress at any of the three post-Katrina surveys (T1, T2, T3). The results for EVI were consistent using 300m and 500m buffers, but at 1000m we did not observe a significant association between change in greenness from baseline to T1 and odds of distress. Finally, as a second sensitivity analysis, we included a binary indicator for whether a person reported any relocation (in lieu of distance of move). The results for were consistent for both EVI and NDVI and across all buffer sizes (e.g., at a 300m buffer we found evidence to support our hypothesis that participants whose neighborhood greenness changed between baseline and T1 from low to high [OR: 3.53, 95% CI: 1.42–8.79] or from high to low [OR: 2.87, 95% CI: 1.15–7.13] had increased odds of psychological distress at T1 compared to participants whose greenness was low at baseline and remained low at T1.

## 4. Discussion

In this study we investigated whether residential greenness was associated with psychological distress among survivors of Hurricane Katrina. Leveraging a unique longitudinal cohort that included pre-disaster data, we tested two hypotheses: 1) residential greenness in post-disaster location was positively associated with mental health, and 2) changes in residential greenness were associated with adverse mental health. We did not find evidence to support our first hypothesis, but did find evidence to support our second hypothesis: participants whose pre-Katrina (T0) and post-Katrina (T1) neighborhoods had different levels of greenness as measured by EVI (*i*.*e*., resided in low greenness neighborhoods pre-storm and high greenness neighborhood post storm or vice versa) had increased odds of distress at T1 compared to those who resided in low greenness neighborhoods both before and after the storm. These findings were not statistically significant with the use of a different measure of greenness (NDVI), indicating that results should be interpreted cautiously. However, while NDVI has historically been the most commonly used metric for quantifying greenness [33], more recent studies apply EVI because it is a potentially more accurate measure. EVI corrects for some distortions in the reflected light, as well as for atmospheric noise and canopy effects [54].

We interpret this result to indicate that a *change* in neighborhood environment, whether due to residential mobility or tree and plant damage from wind and flooding, was associated with psychological distress. This is consistent with the theory of place attachment and indicates that changes in greenness may disrupt sense of place, especially for participants who were displaced by the storm. Nearly all participants relocated at least once between baseline and T1 and for these movers, being displaced to an area with a different residential greenness might contribute to feeling out of place or unconnected with their new residences, contributing to the overall disruption caused by Hurricane Katrina, especially in the short term [2, 36]. Importantly, we adjusted for distance between residential addresses at each survey wave to reduce the possibility that our results are simply capturing the impact of *relocation* on mental health. We also conducted a sensitivity analysis in which we adjusted for a binary indicator of whether a person reported any relocation (in place in distance), and our results were robust to this modification. Additionally, we adjusted for change in NCD to further isolate the impact of greenness from the impact of change in neighborhood more broadly. Ultimately, disentangling different impacts of mobility and neighborhood-level features is difficult and merits future study.

Notably, we did not find convincing evidence of associations between change in greenness (EVI or NDVI) and psychological distress between later survey waves (T1 and T2 nor T2 and T3). We interpret this result as showing that change in greenness might impact mental health in the short-term but not the long-term. Short-term (T1) relocations in the aftermath of Hurricane Katrina were made under duress, often when the possibility of return was limited due to damages that prevented re-occupancy [7]. In these cases, subjects may have had little control over relocation site [55]. In contrast, participants had more control over the timing and destination of later moves. This suggests a new hypothesis: the association between neighborhood greenness and mental health among disaster survivors depends on the degree of control, or lack thereof, that they have in choosing their neighborhood. In the case of non-movers, the full or partial recovery of the natural environment in later survey waves may also diminish this association. However, further research with larger sample sizes is needed to elucidate changes in the relationship between greenness and psychological distress over time.

We did not find evidence to support our first hypothesis: we did not observe an association between cross-sectional greenness (NDVI or EVI) and mental health at any of the survey waves, including before Hurricane Katrina. These findings contrast with existing literature (*i*.*e*., in non-disaster affected populations), which has generally found that residential vegetation is associated with positive mental health [33], including some studies that have specifically assessed psychological distress as measured by K6 [56]. One explanation for this discrepancy is the unique character of the RISK cohort, which is comprised of female, low-income mothers, who may have different underlying mental health statuses. For example, the average K6 score at T0 was 4.68 [SD = 3.68]. This is higher than the mean K6 score for the United States (1999–2017), which ranges from 2.2 to 2.8 [57]. Further, participants in the RISK sample may experience greenness differently than other populations: for example, fear of crime or concerns about managing young children outdoors could present barriers to use of greenspace for this population [58].

These findings also contrast with the only other study, to our knowledge, that assessed the association between neighborhood greenness and mental health in a disaster-affected population. In a cross-sectional study of survivors of Hurricane Harvey (2017) living in Houston, Texas, Li et al. (2021), found that NDVI was not associated with post-traumatic stress disorder ([PTSD] as measured by the Posttraumatic Stress Disorder Checklist for DSM-5), but was associated with reduced post-hurricane distress (as measured by the revised Impact of Event Scale, IES-R) [20]. The data was collected 24–36 months after Hurricane Harvey, making it roughly comparable with T1 in this study (conducted ~1–2 years after Hurricane Katrina). However, as shown in Fig 1, we found no significant association between living in a high vs. low greenness neighborhood at T1 and psychological distress. There are four reasons that our findings may contrast with those of Li et al. First, the RISK cohort is a highly vulnerable group of Hurricane Katrina survivors [59], whereas Li et al.’s study sample was selected to be representative of the population in areas enduring severe loss during Hurricane Harvey. Neighborhood greenness may have a marginal salutary effect on mental health for high-risk subgroups, such as the RISK sample. Second, our study used Kessler’s K6 score while Li et al. (2021) used the IES-R. The IES-R was designed for measuring distress associated with a specific life event [60]. Kessler’s K6 score, in contrast, is a measure of nonspecific psychological distress [37]. Third, our study used MODIS satellite data (250m), while Li et al. used higher-resolution data from the National Agriculture Imagery Program (1m). However, in both studies, NDVI was calculated in residential buffers around the home (300, 500 and 1000m in our study and 1000m in Li et al.). Finally, Hurricanes Katrina and Harvey differed in ways that may affect mental health recovery. Damage to the built environment was far greater in Hurricane Katrina, resulting in displacement of a greater proportion of the population for much longer than in the case of Hurricane Harvey [61, 62]. Whereas nearly 40% of New Orleanians had probable mental illness approximately one year after Hurricane Katrina [10], approximately five months after Hurricane Harvey only 18% of Houston residents reported post-traumatic stress [63].

This study has several limitations. While we were able to follow participants over time, we did not have complete residential histories, even for those who participated in all survey waves. Participants may have moved multiple times between each survey, which was not captured in our exposure assessment (which assessed change between survey waves). This could be more significant for later waves (e.g., between T2 and T3) which were spaced further apart. The chronic strain of multiple relocations may influence mental health outcomes [64], potentially biasing the findings of this study. The impact of repeated relocation after disasters is an important and understudied topic, and one that will become more relevant in the context of global climate change.

Additional limitations include a lack of population representative data and the small sample size of the data. A common limitation for studies of residential greenness is that EVI and NDVI cannot distinguish between beneficial types of green spaces (e.g. urban parks) and markers of neighborhood neglect (e.g. vacant lots) or on the use of and interaction with greenspace. This may be important for our study as land abandonment was one of the primary drivers of post-Katrina changes in vegetation [65]. Future work on greenness and health among disaster survivors should examine different types of vegetation markers, as has recent research on greenness and health in general populations. This could include the use of land-use and land-cover data [66] or street view images [67], to identify between different types of green spaces [68], the quality of those spaces [69, 70], and whether green spaces are accessible [71] and utilized by residents [72]. These approaches could also be used to assess whether greenness is a marker of gentrification, and how changes in neighborhood composition over time impact health outcomes of residents [73]. Future research should also consider individuals’ use of other, non-residential green spaces (e.g., at work or school) [74].

This study also has a number of strengths. We leveraged a unique longitudinal cohort that included pre-disaster data and three waves of post-disaster data. In contrast, much of the literature investigating greenness and health [33] and disasters and health [75] relies on cross-sectional data. The longitudinal design of the RISK study advances knowledge in this field in several ways: first, we were able to control for pre-disaster mental health, thus isolating the effect of the disaster on change in mental health and eliminating the concern that mental health changes were concentrated among those with lower baseline health. Second, this was the first study (to the best of our knowledge) to investigate whether *changes* in greenness were associated with mental health over time. Third, by following participants over nearly 15 years, we could assess the relationship between greenness and health at multiple different time points after Hurricane Katrina.

In conclusion, in this study we investigated whether residential greenness was associated with mental health among survivors of Hurricane Katrina using a unique longitudinal cohort that included pre-disaster data. We found evidence that a *change* in neighborhood greenness (using EVI), whether due to residential mobility or tree and plant damage from wind and flooding, was associated with psychological distress in the short-term. We interpret these findings through the lens of place attachment, positing that the disruption of post-disaster displacement may be attenuated by moves to areas with similar environmental features. We recognize that other factors, such as financial and healthcare support, are likely more important for post-disaster mental health, and do not suggest that residential greenness can outweigh systematic failures to provide adequate protections for those affected by disasters. However, the findings from this study suggest that neighborhood-level environmental features may play have a role to play in supporting the mental health of disaster survivors. Thus, future research on post-disaster neighborhoods, environmental attributes, and place-based attachment could potentially contribute to improve refugee programming and inform additional support for individuals and communities affected by disasters.

## Supporting information

S1 Appendix Appendix containing supporting figures and tables. (DOCX)
