# Supplementary material for: The impact of residential greenness on psychological distress among Hurricane Katrina survivors
Source: PLoS One. 2023 May 11;18(5):e0285510. doi: 10.1371/journal.pone.0285510 (PMC10174552; doi:10.1371/journal.pone.0285510)
Supplement: S1 Appendix — (DOCX) [file pone.0285510.s001.docx]

# S1 Appendix

**S1 Table. Full results for cross-sectional associations (Enhanced Vegetation Index [EVI])** Fully adjusted cross-sectional associations between residential greenness (300m EVI) and odds of psychological distress at each survey wave (n=229). Results from logistic regressions (β [95% CI]) with robust standard errors calculated using Huber-White estimates for all models.

|  | Psychological Distress at Baseline | | Psychological Distress at T1 | | Psychological Distress at T2 | | Psychological Distress at T3 | |
| --- | --- | --- | --- | --- | --- | --- | --- | --- |
| *Predictors* | *Odds Ratios* | *CI* | *Odds Ratios* | *CI* | *Odds Ratios* | *CI* | *Odds Ratios* | *CI* |
| Greenness |  |  |  |  |  |  |  |  |
| Low | Ref |  | Ref |  | Ref |  | Ref |  |
| High | 0.91 | 0.51 - 1.64 | 1.01 | 0.54 - 1.90 | 1.42 | 0.73 - 2.75 | 0.69 | 0.37 - 1.30 |
| NCD |  |  |  |  |  |  |  |  |
| High | Ref |  | Ref |  | Ref |  | Ref |  |
| Low | 0.95 | 0.52 - 1.75 | 0.52 | 0.27 - 0.97 | 1.53 | 0.78 - 3.0 | 0.99 | 0.52 - 1.90 |
| Mental Distress at Previous Wave |  |  |  |  |  |  |  |  |
| No | - | - | Ref |  | Ref |  | Ref |  |
| Yes |  |  | 1.25 | 1.13 - 1.37 | 1.27 | 1.16 - 1.39 | 1.07 | 1.01 - 1.15 |
| Age (1 year increment) | 0.98 | 0.91 - 1.05 | 1.1 | 1.02 - 1.19 | 0.95 | 0.88 - 1.03 | 1.03 | 0.96 - 1.10 |
| Race |  |  |  |  |  |  |  |  |
| White | Ref |  | Ref |  | Ref |  | Ref |  |
| Black | 1.76 | 0.59 - 5.25 | 0.79 | 0.23 - 2.68 | 0.76 | 0.21 - 2.72 | 0.69 | 0.24 - 2.00 |
| Other | 1.29 | 0.27 - 6.14 | 0.43 | 0.08 - 2.29 | 0.76 | 0.1 - 5.69 | 1.84 | 0.33 - 10.16 |
| Current^[[1]](#endnote-1)^ level of social support  (scale 1-4) | 0.19 | 0.09 - 0.39 | 0.59 | 0.3 - 1.19 | 0.26 | 0.12 - 0.61 | 0.38 | 0.2 - 0.74 |
| Currently receiving benefits |  |  |  |  |  |  |  |  |
| Yes | Ref |  | Ref |  | Ref |  | Ref |  |
| No | 1.72 | 0.84 - 3.49 | 1.22 | 0.55 - 2.71 | 0.44 | 0.22 - 0.89 | 0.52 | 0.27 - 0.97 |
| Currently living in New Orleans |  |  |  |  |  |  |  |  |
| No | - | - | Ref |  | Ref |  | Ref |  |
| Yes | - | - | 0.64 | 0.26 - 1.59 | 1.4 | 0.71 - 2.79 | 1.1 | 0.55 - 2.22 |
| Experienced significant damage during Katrina |  |  |  |  |  |  |  |  |
| No | - | - | Ref |  | Ref |  | Ref |  |
| Yes | - | - | 0.74 | 0.38 - 1.46 | 1.2 | 0.6 - 2.37 | 0.76 | 0.39 - 1.51 |
| Distance of move (km) | - | - | 1.10 | 1.05 - 1.15 | 1.00 | 0.99 - 1.00 | 1.02 | 1.01-1.05 |

**S2 Table. Full results for cross-sectional associations (Normalized Difference Vegetation Index [NDVI])** Fully adjusted cross-sectional associations between residential greenness (300m NDVI) and odds of psychological distress at each survey wave (n=229). Results from logistic regressions (β [95% CI]) with robust standard errors calculated using Huber-White estimates for all models.

|  | Psychological Distress at Baseline | | Psychological Distress at T1 | | Psychological Distress at T2 | | Psychological Distress at T3 | |
| --- | --- | --- | --- | --- | --- | --- | --- | --- |
| *Predictors* | *Odds Ratios* | *CI* | *Odds Ratios* | *CI* | *Odds Ratios* | *CI* | *Odds Ratios* | *CI* |
| Greenness |  |  |  |  |  |  |  |  |
| Low | Ref |  | Ref |  | Ref |  | Ref |  |
| High | 0.96 | 0.54 - 1.72 | 1.15 | 0.61 - 2.18 | 1.33 | 0.69 - 2.58 | 0.92 | 0.49 - 1.71 |
| NCD |  |  |  |  |  |  |  |  |
| High | Ref |  | Ref |  | Ref |  | Ref |  |
| Low | 0.95 | 0.52 - 1.75 | 0.51 | 0.27 - 0.96 | 1.51 | 0.77 - 2.97 | 1.01 | 0.53 - 1.96 |
| Mental Distress at Previous Wave |  |  |  |  |  |  |  |  |
| No | - | - | Ref |  | Ref |  | Ref |  |
| Yes |  |  | 1.24 | 1.13 - 1.37 | 1.27 | 1.16 - 1.39 | 1.08 | 1.01 - 1.15 |
| Age (1 year increment) | 0.98 | 0.91 - 1.05 | 1.1 | 1.02 - 1.18 | 0.95 | 0.88 - 1.03 | 1.03 | 0.96 - 1.10 |
| Race |  |  |  |  |  |  |  |  |
| White | Ref |  | Ref |  | Ref |  | Ref |  |
| Black | 1.75 | 0.59 - 5.24 | 0.8 | 0.24 - 2.68 | 0.78 | 0.22 - 2.79 | 0.74 | 0.26 - 2.16 |
| Other | 1.26 | 0.26 - 5.99 | 0.44 | 0.08 - 2.32 | 0.74 | 0.1 - 5.58 | 1.9 | 0.36 - 10.18 |
| Current^[[2]](#endnote-2)^ level of social support  (scale 1-4) | 0.19 | 0.09 - 0.39 | 0.59 | 0.3 - 1.19 | 0.27 | 0.12 - 0.62 | 0.38 | 0.2 - 0.73 |
| Currently receiving benefits |  |  |  |  |  |  |  |  |
| Yes | Ref |  | Ref |  | Ref |  | Ref |  |
| No | 1.72 | 0.85 - 3.5 | 1.24 | 0.56 - 2.75 | 0.44 | 0.21 - 0.88 | 0.51 | 0.27 - 0.96 |
| Currently living in New Orleans |  |  |  |  |  |  |  |  |
| No | - | - | Ref |  | Ref |  | Ref |  |
| Yes | - | - | 0.64 | 0.25 - 1.59 | 1.4 | 0.7 - 2.79 | 1.1 | 0.54 - 2.22 |
| Experienced significant damage during Katrina |  |  |  |  |  |  |  |  |
| No | - | - | Ref |  | Ref |  | Ref |  |
| Yes | - | - | 0.75 | 0.38 - 1.46 | 1.21 | 0.61 - 2.41 | 0.76 | 0.39 - 1.5 |
| Distance of move (km) | - | - | 1.01 | 1.05 - 1.15 | 1.00 | 0.99 - 1.00 | 1.02 | 1.01-1.05 |

**S3 Table. Full results for change in greenness (Enhanced Vegetation Index [EVI])** Association between change in greenness (EVI) between survey waves and odds of psychological distress at each survey wave, controlling for psychological distress at the previous wave (n=229). Results from logistic regressions (β [95% CI]) with robust standard errors calculated using Huber-White estimates for all models.

|  | Psychological Distress at T1  (distress vs. no distress) | | Psychological Distress at T2  (distress vs. no distress) | | Psychological Distress at T3  (distress vs. no distress) | |
| --- | --- | --- | --- | --- | --- | --- |
| *Predictors* | *Odds Ratio* | *CI* | *Odds Ratio* | *CI* | *Odds Ratio* | *CI* |
| Greenness (comparing current to previous wave) |  |  |  |  |  |  |
| Low-low | Ref |  | Ref |  | Ref |  |
| Low-high | 3.48 | 1.40 - 8.62 | 1.81 | 0.73 - 4.52 | 1.38 | 0.54 - 3.57 |
| High-low | 2.60 | 1.05 - 6.42 | 1.97 | 0.76 - 5.10 | 0.48 | 0.19 - 1.24 |
| High-high | 1.82 | 0.74 - 4.51 | 2.23 | 0.88 - 5.62 | 1.23 | 0.52 - 2.94 |
| NCD (comparing current to previous wave) |  |  |  |  |  |  |
| High-high | Ref |  | Ref |  | Ref |  |
| High-low | 0.87 | 0.31 - 2.44 | 0.7 | 0.24 - 2.03 | 0.61 | 0.24 - 1.53 |
| Low-high | 1.12 | 0.45 - 2.78 | 0.79 | 0.25 - 2.46 | 0.75 | 0.30 - 1.88 |
| Low-low | 1.93 | 0.83 - 4.46 | 0.44 | 0.20 - 0.96 | 0.77 | 0.29 - 1.99 |
| Mental Distress at Previous Wave |  |  |  |  |  |  |
| No | Ref |  | Ref |  | Ref |  |
| Yes | 2.92 | 1.46 - 5.83 | 5.23 | 2.62 - 10.45 | 2.01 | 1.05 - 3.83 |
| Age (1 year increment) | 1.08 | 1 - 1.16 | 0.96 | 0.89 - 1.03 | 1.01 | 0.94 - 1.09 |
| Race |  |  |  |  |  |  |
| White | Ref |  | Ref |  | Ref |  |
| Black | 0.63 | 0.18 - 2.18 | 0.91 | 0.24 - 3.42 | 0.71 | 0.24 - 2.13 |
| Other | 0.45 | 0.08 - 2.46 | 0.78 | 0.12 - 5.08 | 1.61 | 0.29 - 8.82 |
| Current^[[3]](#endnote-3)^ level of social support  (scale 1-4) | 0.57 | 0.29 - 1.12 | 0.27 | 0.12 - 0.58 | 0.34 | 0.17 - 0.69 |
| Currently receiving benefits |  |  |  |  |  |  |
| Yes | Ref |  | Ref |  | Ref |  |
| No | 1.2 | 0.54 - 2.67 | 0.41 | 0.2 - 0.82 | 0.5 | 0.26 - 0.97 |
| Currently living in New Orleans |  |  |  |  |  |  |
| No | Ref |  | Ref |  | Ref |  |
| Yes | 0.59 | 0.26 - 1.34 | 1.57 | 0.77 - 3.18 | 1.03 | 0.49 - 2.16 |
| Experienced significant damage during Katrina |  |  |  |  |  |  |
| No | Ref |  | Ref |  | Ref |  |
| Yes | 0.7 | 0.36 - 1.38 | 1.06 | 0.55 - 2.07 | 0.77 | 0.38 - 1.57 |
| Distance of move (km) | 1.12 | 1.06 - 1.16 | 1.01 | 1.00 - 1.12 | 1.03 | 0.98 - 1.04 |

**S4 Table. Full results for change in greenness associations (Normalized Difference Vegetation Index [NDVI])** Association between change in greenness (NDVI) between survey waves and odds of psychological distress at each survey wave, controlling for psychological distress at the previous wave (n=229). Results from logistic regressions (β [95% CI]) with robust standard errors calculated using Huber-White estimates for all models.

|  | Psychological Distress at T1  (distress vs. no distress) | | Psychological Distress at T2  (distress vs. no distress) | | Psychological Distress at T3  (distress vs. no distress) | |
| --- | --- | --- | --- | --- | --- | --- |
| *Predictors* | *Odds Ratio* | *CI* | *Odds Ratio* | *CI* | *Odds Ratio* | *CI* |
| Greenness (comparing current to previous wave) |  |  |  |  |  |  |
| Low-low | Ref |  | Ref |  | Ref |  |
| Low-high | 1.94 | 0.82 - 4.57 | 1.65 | 0.64 - 4.25 | 1.23 | 0.49 - 3.11 |
| High-low | 1.87 | 0.78 - 4.48 | 1.91 | 0.73 - 4.95 | 0.61 | 0.24 - 1.53 |
| High-high | 1.79 | 0.75 - 4.27 | 1.9 | 0.7 - 5.16 | 1.57 | 0.64 - 3.87 |
| NCD (comparing current to previous wave) |  |  |  |  |  |  |
| High-high | Ref |  | Ref |  | Ref |  |
| High-low | 1.00 | 0.35 - 2.8 | 0.71 | 0.24 - 2.11 | 0.61 | 0.24 - 1.54 |
| Low-high | 1.26 | 0.52 - 3.07 | 0.8 | 0.26 - 2.48 | 0.7 | 0.27 - 1.78 |
| Low-low | 2.24 | 0.97 - 5.16 | 0.46 | 0.21 - 1.01 | 0.75 | 0.29 - 1.94 |
| Mental Distress at Previous Wave |  |  |  |  |  |  |
| No | Ref |  | Ref |  | Ref |  |
| Yes | 2.72 | 1.37 - 5.39 | 5.2 | 2.63 - 10.28 | 2.08 | 1.1 - 3.93 |
| Age (1 year increment) | 1.08 | 1 - 1.16 | 0.96 | 0.89 - 1.03 | 1.02 | 0.94 - 1.09 |
| Race |  |  |  |  |  |  |
| White | Ref |  | Ref |  | Ref |  |
| Black | 0.64 | 0.2 - 1.99 | 0.91 | 0.24 - 3.44 | 0.81 | 0.28 - 2.38 |
| Other | 0.51 | 0.1 - 2.57 | 0.76 | 0.12 - 4.87 | 1.64 | 0.31 - 8.69 |
| Current^[[4]](#endnote-4)^ level of social support  (scale 1-4) | 0.53 | 0.28 - 1.02 | 0.28 | 0.13 - 0.61 | 0.35 | 0.17 - 0.69 |
| Currently receiving benefits |  |  |  |  |  |  |
| Yes | Ref |  | Ref |  | Ref |  |
| No | 1.2 | 0.56 - 2.6 | 0.4 | 0.2 - 0.8 | 0.48 | 0.25 - 0.93 |
| Currently living in New Orleans |  |  |  |  |  |  |
| No | Ref |  | Ref |  | Ref |  |
| Yes | 0.61 | 0.26 - 1.43 | 1.55 | 0.77 - 3.12 | 1.03 | 0.49 - 2.16 |
| Experienced significant damage during Katrina |  |  |  |  |  |  |
| No | Ref |  | Ref |  | Ref |  |
| Yes | 0.77 | 0.4 - 1.48 | 1.08 | 0.55 - 2.12 | 0.8 | 0.39 - 1.61 |
| Distance of move (km) | 1.10 | 1.05 - 1.16 | 1.05 | 1.04 - 1.13 | 1.03 | 0.99 - 1.05 |

**S1 Fig. Sensitivity analysis at different spatial buffers.** Fully adjusted cross-sectional associations between residential greenness (high vs. low greenness areas, measured by NDVI and EVI 300m buffers) and odds of psychological distress (K6 ≥ 5) at each survey wave (n=229). Results from multivariable logistic regressions (β [95% CI]) with robust standard errors calculated using Huber-White estimates for all models.


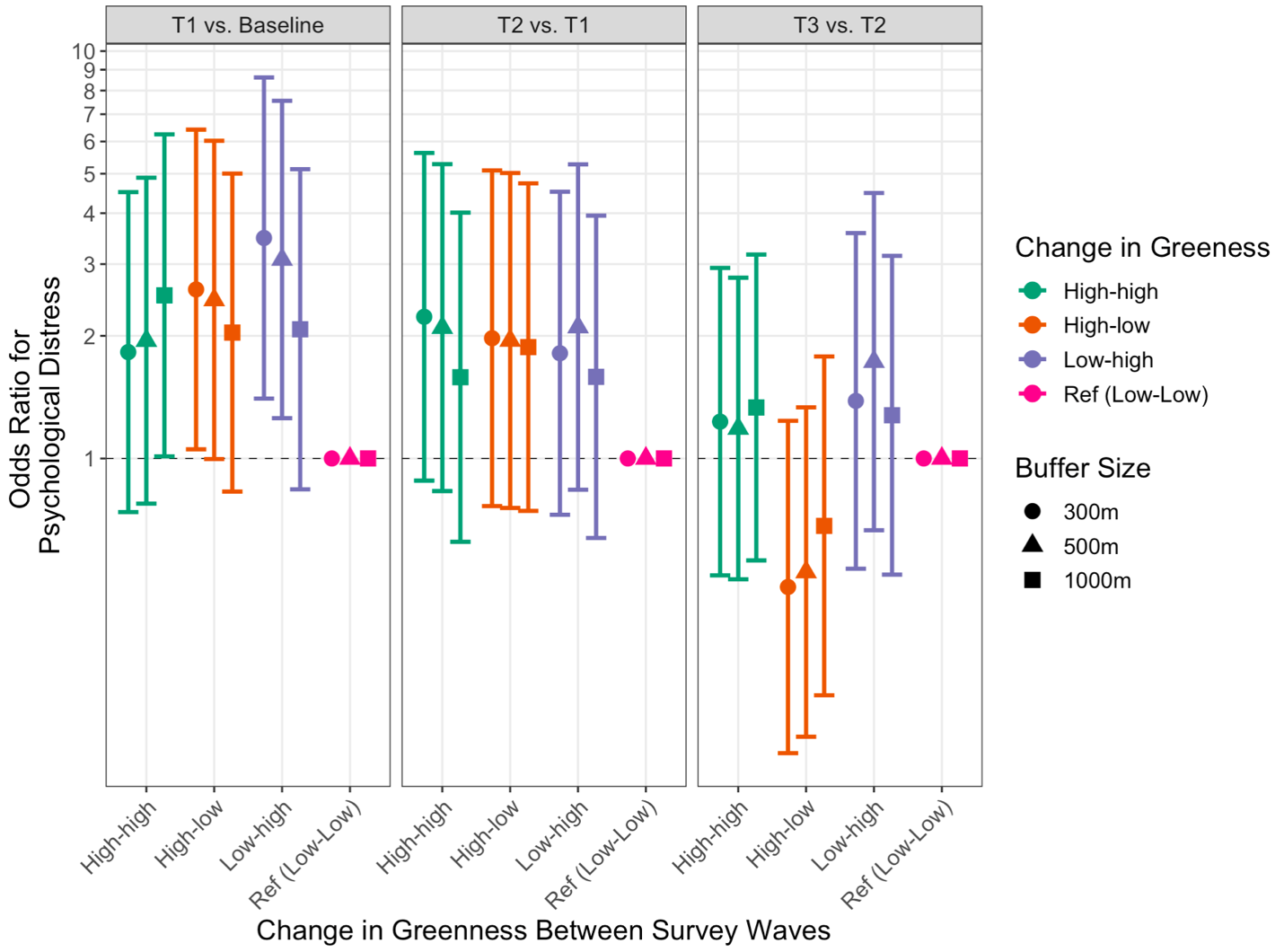


1. All variables labeled “current” refer to status of that variable at the time of the survey wave corresponding to each column. For example, in the column “Psychological Distress at T1,” the level of social support refers to the level of social support at T1. [↑](#endnote-ref-1)
2. All variables labeled “current” refer to status of that variable at the time of the survey wave corresponding to each column. For example, in the column “Psychological Distress at T1,” the level of social support refers to the level of social support at T1. [↑](#endnote-ref-2)
3. All variables labeled “current” refer to status of that variable at the time of the survey wave corresponding to each column. For example, in the column “Psychological Distress at T1,” the level of social support refers to the level of social support at T1. [↑](#endnote-ref-3)
4. All variables labeled “current” refer to status of that variable at the time of the survey wave corresponding to each column. For example, in the column “Psychological Distress at T1,” the level of social support refers to the level of social support at T1. [↑](#endnote-ref-4)
